# The Effect of Perturbation-Based Balance Training and Conventional Intensive Balance Training on Reactive Stepping Ability in Individuals With Incomplete Spinal Cord Injury or Disease: A Randomized Clinical Trial

**DOI:** 10.3389/fneur.2021.620367

**Published:** 2021-02-02

**Authors:** Janelle Unger, Katherine Chan, Jae W. Lee, B. Catharine Craven, Avril Mansfield, Mohammad Alavinia, Kei Masani, Kristin E. Musselman

**Affiliations:** ^1^Rehabilitation Sciences Institute, University of Toronto, Toronto, ON, Canada; ^2^KITE at Toronto Rehabilitation Institute-University Health Network, Toronto, ON, Canada; ^3^Institute of Biomedical Engineering, University of Toronto, Toronto, ON, Canada; ^4^Department of Medicine, Division of Physical Medicine and Rehabilitation, University of Toronto, Toronto, ON, Canada; ^5^Institute of Health Policy, Management and Evaluation, University of Toronto, Toronto, ON, Canada; ^6^Evaluative Clinical Sciences, Hurvitz Brain Sciences Program, Sunnybrook Research Institute, Toronto, ON, Canada; ^7^Department of Physical Therapy, University of Toronto, Toronto, ON, Canada

**Keywords:** spinal cord injury, balance, rehabilitation, postural control, fall prevention, randomized clinical trial

## Abstract

**Introduction:** Impaired balance leads to falls in individuals with motor incomplete spinal cord injury or disease (iSCI/D). Reactive stepping is a strategy used to prevent falls and Perturbation-based Balance Training (PBT) can improve this ability.

**Objective:** The objective of this study was to determine if PBT results in greater improvements in reactive stepping ability than frequency-matched Conventional Intensive Balance Training (CIBT) in adults with iSCI/D.

**Design:** Randomized clinical trial.

**Setting:** Tertiary SCI/D rehabilitation center.

**Participants:** Twenty-one adults with chronic (>1 year) iSCI/D were randomized. Due to one drop out 20 participants completed the study.

**Methods:** Participants were randomly allocated to complete either PBT or CIBT three times per week for 8 weeks. Both programs included challenging static and dynamic balance tasks, but the PBT group also experienced manual external balance perturbations.

**Main Outcome Measures:** Assessments of reactive stepping ability using the Lean-and-Release test were completed at baseline, and after 4 and 8 weeks of training, and 3 and 6 months after training completion. A blinded assessor evaluated secondary outcomes.

**Results:** Twenty-five participants were screened and 21 consented; one withdrew. Ten PBT and 10 CIBT participants were included in analyses. Across all participants there were improvements in reactive stepping ability (*p* = 0.049), with retention of improvements at follow up assessments. There were no differences in reactive stepping ability between groups [median (interquartile range): PBT 0.08 (0.68); CIBT 0.00 (0.22)]. One participant in the PBT group experienced a non-injurious fall during training.

**Conclusions:** Balance training is beneficial for individuals with iSCI/D, but the addition of manual perturbations (i.e., PBT) did not prove advantageous for performance on a measure of reactive stepping ability.

**Clinical Trial Registration:** www.ClinicalTrials.gov, identifier: NCT02960178.

## Introduction

Each year ~78% of ambulatory individuals with motor incomplete spinal cord injury or disease (iSCI/D) fall (1). Falls can have negative consequences, including physical injury (1), hospital admissions (2), or reduced participation in physical activity (3), as well as psychosocial consequences, such as embarrassment, social isolation, decreased participation in the community, and fear of falling (1). Although impaired balance is a leading cause of falls in this population (4) the current time in physical therapy allocated to balance training is limited (5). This is despite the fact that time spent on balance training during inpatient rehabilitation increases the odds of being ambulatory at discharge in people with motor incomplete SCI/D (iSCI/D) (5). Research on balance training after a motor iSCI/D is also limited (6); the majority of published studies assessing changes in balance do so after locomotor training, rather than a balance intervention (7–12). Previously studied balance interventions have focused on anticipatory balance control, which is used to stabilize the body before a volitional movement (13). For example, using visual feedback during standing balance tasks (14, 15), virtual reality (16, 17), or a divided-attention stepping task (18) that focuses on voluntary movements to train balance. Balance studies in iSCI/D research are often pre-post analyses (14, 16, 17), include retrospective controls (15), or are case studies (18), which are considered lower levels of evidence than randomized trials. Furthermore, studies that do use a balance-focused intervention typically only include outcomes that measure standing balance (14, 15, 18), even though most falls experienced by ambulatory individuals with iSCI/D occur during walking (4).

Balance is lost when the individual loses control of the relationship between the center of mass and the base of support. Anticipatory strategies are used to prevent balance loss, whereas reactive strategies are used to prevent a fall following a loss of balance (13). Reactive stepping is a specific reactive balance control strategy where the individual takes a step to increase the size of the base of support and uses the force at step contact to decelerate the falling center of mass (13). Impaired reactive stepping ability, as evidenced by delayed stepping responses or an increased number of steps or assistance needed to recover balance, leads to increased falls in people with stroke and Parkinson's disease (19, 20). People with chronic iSCI/D take more steps to recover from a balance perturbation than age- and sex-matched able-bodied individuals, indicating impaired reactive stepping ability (21). However, individuals with iSCI/D did not show delayed stepping responses when compared to individuals without SCI/D (21). Reactive balance is not regularly trained in current rehabilitation practices (22), but previous research has demonstrated the value of training reactive balance in people at increased risk of falling (23). Perturbation-based Balance Training (PBT) is an intervention used to train reactive stepping ability (24). During PBT, participants experience repeated balance perturbations (~1 perturbation per minute, although the optimal dose has yet to be determined) during conventional mobility related activities, such as standing and walking (24). These balance perturbations are designed to evoke reactive step(s), providing opportunities for participants to practice and improve control of these steps (24). Following up to 24 PBT sessions, individuals with Parkinson's disease or stroke have improved reactive stepping ability (25), balance outcomes (26, 27), increased balance confidence (26), and decreased fall rates (23, 28, 29). Reactive strategies occur faster than volitional movements but slower than short-loop reflexes and are thought to be controlled through long-loop reflexes (30, 31). Although, there is likely cortical involvement in reactive stepping (30, 31), the role of the spinal cord in the response may suggest that people with iSCI/D will respond differently compared to individuals with stroke or Parkinson's disease.

The primary objective of this study was to determine if PBT results in greater improvements in reactive stepping ability, measured by the number of steps required to recover, when compared with a frequency-matched, conventional approach to balance training (Conventional Intensive Balance Training; CIBT). Secondary objectives were to compare performance on clinical measures of balance, strength, and gait between the two balance training methods, as well as self-reported balance confidence and fall concern. We also compared the effects of the two balance training methods on the number of participants who experienced falls during the follow-up period, as well as the number of falls and time to first fall. We hypothesized that more participants in the PBT group would demonstrate improvements in reactive stepping ability, as well as the other balance, strength, gait, and self-report measures, following training when compared to the CIBT group, as PBT has been shown to improve these outcomes (26, 27), and that these improvements would be retained at 3 and 6 months after training completion. We also hypothesized that fewer participants who completed PBT would experience a fall, and the PBT group would experience fewer total falls as well as a longer time to first fall when compared to CIBT.

## Materials and Methods

### Trial Design

This study was a single-site, assessor blinded, randomized clinical trial (RCT) (ClinicalTrials.gov identifier NCT02960178) that took place at the Toronto Rehabilitation Institute–University Health Network (UHN). Ethical approval was obtained by the Research Ethics Board of the UHN (study ID: 16-5685) and all participants provided informed consent before beginning the study. A detailed version of this protocol is available (32), but a brief outline is presented here.

### Participants

Participants were adults with a non-progressive motor iSCI/D [i.e., American Spinal Injury Association Impairment Scale (AIS) rating of C or D according to the International Standards for Neurological Classification of Spinal Cord Injury (33)] of traumatic or non-traumatic etiology. All participants were at least 1 year post-injury, after which natural recovery is minimal (34), were able to stand independently for at least 30 s and demonstrated a moderate level of trunk control, determined by the ability to reach forward 2 inches with an outstretched arm while standing unsupported (35). Participants were excluded if their participation in the study could be affected by spasticity, contractures, pressure sores, cardiac conditions, or comorbidities (32). Participants were asked not to begin any new rehabilitation or exercise programs during their time in the study, however in the case we became aware of participation in a new program these details were noted.

### Outcomes

Six assessments were completed; two baseline assessments, a midpoint assessment (4-week), a final assessment (8-week), and two follow-up assessments at 3 and 6 months post-training (32). As described previously (32), the Lean-and-Release test is a standardized method of assessing reactive balance by simulating a forward fall. The Lean-and-Release test was conducted by the research team, who were not blinded to participant allocation, due to feasibility, as the assessment requires three individuals with technical training. To induce a forward fall, we used a horizontal tether at the level of the sacrum attached behind the participant [refer to (21) for description]. The tether was attached to a force transducer allowing measurement of the amount of body weight supported by the tether, as well as recording the time of the unexpected release. Two dual force plates measuring 502 × 502 millimeters were used (Advanced Mechanical Technology Inc., Watertown, USA), with participants standing with one leg each on the posterior plates with the other force plates positioned directly anterior. The force plates were built into a wooden platform, creating a flat surface that was accessible by a ramp. Data were collected using PowerLab DAQ (ADInstruments Inc., Colorado Springs, Colorado, USA) and Lab Chart 7 (ADInstruments Inc., Colorado Springs, Colorado, USA) at a frequency of 2,000 Hz, and analog signals from the force transducer and force plates were amplified and converted analog-to-digital (A/D) using a 16-bit A/D card (±5 volts input range National Instruments, Austin, Texas, USA). The tether was not released unless the participants had between 8 and 12% of their body weight supported through it, and participants were instructed to recover their balance through whatever means necessary, which was expected to be stepping, given the magnitude of the perturbation (21). Participants completed up to 10 trials according to their tolerance, with three false trials interspersed to mitigate the use of anticipatory strategies for a total of 13 trials on six occasions.

From the Lean-and-Release test the behavioral response, as well as foot contact time, were collected. Behavioral response, which was the primary outcome for this study, was calculated as the proportion of trials that participants were able to complete a single reactive step. Recovering with a single step is considered successful (36), and the perturbation was an appropriate magnitude (i.e., ~10% of the bodyweight supported through the tether) to consistently elicit single step responses in able-bodied individuals (21). If the participant required multiple steps or assistance from a spotter or safety harness to recover the trial was deemed unsuccessful. The behavioral response to the Lean-and-Release test was chosen as our primary outcome as it has previously been used in research characterizing reactive stepping in healthy young adults (37), and improved following PBT in adults who experienced a stroke (25). Other studies using PBT have also shown improvements in the number of reactive steps used to recover balance (27, 38). Foot contact time was the time taken for participants to exert >1% of the body weight on the force plate with the stepping leg following the release. Foot contact time was only calculated for the first step, even if multiple steps were taken following the release (21). Both of these variables have good test-retest reliability, and the behavioral responses have demonstrated convergent validity with measures of lower extremity strength and balance confidence in this population (39).

Secondary outcome measures of balance and strength were completed by one of two assessors blind to group allocation; the blinded assessor was consistent within participants. Measures included the Mini Balance Evaluation Systems Test (Mini-BESTest) (40), the Community Balance and Mobility Scale (CB&M) (41), and lower extremity manual muscle testing (LE MMT) (42) of 12 muscles groups bilaterally (hip flexors, extensors, adductors, abductors, internal rotators, external rotators, knee flexors and extensors, ankle dorsiflexors and plantarflexors, invertors, and evertors). Each muscle group was scored out a possible five points, with zero indicating no muscle contraction and five indicating full strength, with half points allotted for plus/minus scores (42). This scoring resulted in a possible total of 120 points (60 per limb). The CB&M measures a higher level of balance ability than the Mini-BESTest. Gait parameters, including step length, walking speed, cadence, and double support percentage, were collected by a researcher at each assessment using the Zeno Walkway (Model 485, Ver. J, Prokinetics, Havertown, Pennsylvania, USA). Participants completed two passes of the walkway at a self-selected speed using a gait aid if necessary. Each of these measures has been validated for use or has been previously used in iSCI/D research, and our protocol outlines each of these measures in further detail (32).

Self-report measures included the Activities-specific Balance Confidence (ABC Scale) (43) and the Falls Efficacy Scale—International (FES-I) (44), which evaluate balance confidence and fall concern, respectively. The ABC Scale is a self-report measure where participants rate their confidence in performing daily tasks during standing and walking using percentages, with higher percentages indicating more confidence (43). The FES-I is a self-report measure evaluating the level of concern participants have in regards to falling during daily tasks, which is used as a proxy for fear of falling (44).

During the 6-month follow-up period the number of falls experienced by participants were tracked. Participants completed a fall survey, either on paper or online (Qualtrics® Software), within 24 h of experiencing a fall, defined as “inadvertently coming to rest on the ground, floor, or other lower level” (45). The fall survey was based on one previously used in research (46). The survey consisted of closed-ended multiple choice questions about the time of day and location of the fall, and multiple choice questions with an open-ended “other” option about the activity being performed when the fall occurred along with possible contributing factors for the fall. To ensure the fall surveys were completed, participants were interviewed every 3 weeks by a researcher.

### Interventions

Participants were randomized into two equal groups using blocked randomization (block size four). Opaque envelopes were used to randomize participants by someone not involved in study procedures. Both groups completed individualized, challenging static and dynamic balance tasks. The PBT group also experienced manual pushes and pulls from one of four trained members of the research team with a background in either physical therapy or kinesiology. The manual pushes and pulls were delivered approximately once per minute throughout each session, which was based on previous literature (27). The intensity of the manual pushes and pulls was determined by the ability of the participant to withstand the perturbation and progressed as able throughout the program (i.e., if the participant was able to recover without a step the intensity was increased). The balance training tasks were individualized for each participant, and the participant rated the level of challenge after each activity (7) on an 11-point ordinal scale (0 = very easy, 10 = very challenging—would fall without assistance) to ensure the tasks were challenging (i.e., ≥7) (32). Balance training tasks fell under five categories: (1) stable, (2) quasi-mobile, (3) mobile, (4) unpredictable, or (5) participant-selected. Within each category various tasks were recommended, with modifications to increase or decrease difficulty added during the individual sessions (32). These categories and tasks have been modified from other programs offering PBT (27). Rest breaks were offered throughout the session as requested by the participant or to allow for equipment setup. Step reactions (i.e., single step, multi-step, or assistance required to prevent a fall) were also recorded during the sessions, for both groups (32). The balance training programs were completed while wearing a safety harness that allowed free movement within a two meter by four meter frame, although for some participants the time spent in the harness was gradually reduced, or it was taken off for activities completed outside of the frame (i.e., outdoor mobility) (32). Both groups received a higher frequency of balance training than what is typically received, even during inpatient rehabilitation (5). Participants attended PBT or CIBT for 1 hour, three times per week for 8 weeks (32), providing a total of 24 h of balance training.

### Sample Size

The sample size calculation was based on the primary outcome, the behavioral response during the Lean-and-Release test. As previously described (32), baseline data from 11 participants were used to determine how many participants would be required to see a clinically relevant change (≥0.50), determined by the authors, on the proportion of successfully completed trials. Based on this calculation, we determined that 11 participants per group was the target sample size. An *ad hoc* interim analysis using the baseline and post-training data from 17 participants was subsequently performed. The decision to complete this analysis was made based on the observation that the two treatment groups were demonstrating similar improvements in the primary and secondary outcomes. Chi Square tests of independence comparing the number of participants who achieved a clinically significant change on the primary outcome indicated no difference (χ^2^ = 11.27, *p* = 0.34). Based on these results, we re-calculated the sample size using the observed difference in outcomes between groups rather than the desired difference, which indicated that a significant between-group difference on our primary outcome would be obtained with a sample size of 1,568, suggesting no true difference between groups on the primary outcome. Therefore, we completed the study with all the participants who had consented to participate at the time of the interim analysis, 10 in each group, and closed recruitment.

### Data Analysis

*T*-tests were used to compare baseline scores between groups. For the primary and secondary outcome measures, the average value of the two baseline scores was calculated, and descriptive data, including mean (standard deviation) or median (IQR) for change scores were calculated depending on normality. Data were analyzed using intention-to-treat and complete case analysis (47). The mean (standard deviation) of the step reactions implemented during training were calculated for both groups and an independent *t*-test was used to confirm the PBT group experienced more step reactions (single step, multi-steps, or “falls”) during training. A two-way mixed ANOVA was used to determine if challenge ratings changed over time between the third, twelfth, and final training session (within-subject) or were different between groups (between subjects).

To compare groups across time, from baseline to 8-week, a two-way mixed ANOVA was used for all outcome measures regardless of normality as ANOVA calculations have proven to be robust when the assumption of normality is violated, even in small sample sizes (48). Three levels of time, the baseline average, 4-week, and 8-week scores were used to measure effects of time as a within-subject factor and group (i.e., PBT or CIBT) as a between-subjects factor. Bonferroni pairwise comparisons were used to determine the effects between multiple timepoints.

For the primary outcome, a Chi Square test of independence was completed to compare between groups the number of participants who surpassed a clinically significant change of 0.5 for the proportion of trials demonstrating a single step. Chi Square tests of independence were also used to compare between groups the number of participants who surpassed the minimal detectable change (MDC) on the Mini-BESTest (5 points), CB&M (13 points), step length (0.17 m), walking speed (0.17 m/s), cadence (13 steps/min), the ABC Scale (15%). No MDC has been established for the FES-I, but a cut-off score of 22 points has been identified for risk of falling in older adults (49), so a Chi Square test of independence was used to compare those who were above and below this cutoff score. No Chi Square test of independence was performed for the LE MMT or double support percentage of gait, as no MDC for the iSCI/D population has been defined for these outcome measures.

Gardner-Altman estimation plots, which show the observed values of both groups, mean or median data, as well as effect size with a 95% confidence interval, were developed for the measures which showed change over time, using Ho et al. (50). To measure the retention of training effects, a two-way mixed ANOVA was used to compare baseline, final, and 3 month follow up visit scores.

Fall circumstances were reported descriptively. A Chi Square test of independence was used to determine if the number of people experiencing at least one fall, number of people who were frequent fallers (i.e., ≥2), or number of injurious falls differed between groups, and a Poisson regression model was conducted to determine if the number of falls differed between groups. For number of people experiencing at least one fall a relative risk calculation was performed, and for number of falls an incident rate calculation was done. A Kaplan-Meier analysis was done to determine if time to first fall differed between groups. Alpha was set to 0.05, and all statistical analyses were done using SPSS (IBM Corporation, Version 26).

## Results

### Participants, Training, and Adherence

**Figure 1:**
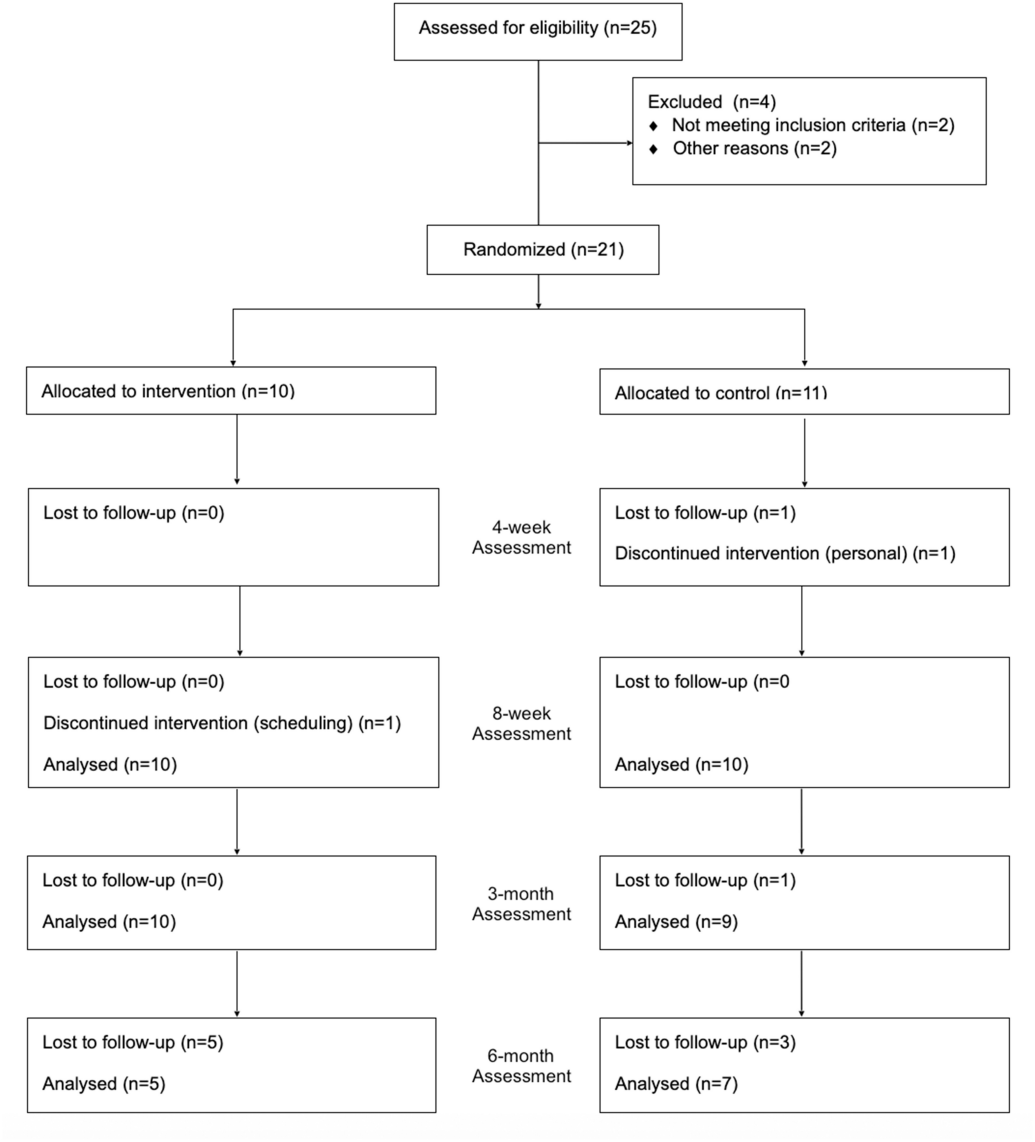
CONSORT flow diagram depicting the flow of participants through the study.

A total of 21 adults with iSCI participated in this study (see Figure 1) from February 2017 to August 2019. One participant allocated to the CIBT group withdrew after 12 training sessions and was unavailable for subsequent assessments. The baseline characteristics of the 20 participants who completed the study are presented in Table 1, there were no differences between the group characteristics at baseline. One participant in the PBT group withdrew after 15 training sessions due to having too many other appointments, but completed all assessments and was analyzed as intention-to-treat. Six CIBT and seven PBT participants were able to progress to spending time outside of the harness during training. Data from two participants, one per group, were not available for the 4-week Lean-and-Release test. One data set was lost due to equipment errors and the other was not completed due to scheduling constraints (i.e., participant had limited availability over the December holiday period).

**Table 1:** Participant demographics by group and total sample.

**Group**	**PBT (*n* = 10)**	**CIBT(*n* = 10)**	**Total Sample (*n* = 20)**
Age (years)	57.9 (±14.2)	55.8 (±15.3)	56.9 (±14.4)
Sex (number)			
Female	7	6	13
Male	3	4	7
Level of injury (number)			
Cervical	3	7	10
Thoracic	5	3	8
Lumbar	2		2
Time since injury (months)	66.5 (±56.8)	114.3 (±144.6)	90.4 (±109.7)
Mechanism of injury (number)			
Traumatic	3	4	7
Non-traumatic	7	6	13
Mobility			
Walking unaided	5	5	10
Walking with aid	2	2	4
Wheelchair user	3	3	6
Behavioral response score (/1)	0.33 (±0.38)	0.25 (±0.33)	0.29 (±0.35)
Mini-BESTest score (/28)	15.1 (±9.3)	11.5 (±8.9)	13.3 (±9.0)
LE MMT score (total /120)	87.75 (±14.66)	84.43 (±11.90)	86.09 (±13.11)
R leg (/60)	44.08 (±6.47)	41.73 (±7.90)	42.90 (±7.13)
L leg (/60)	43.68 (±8.70)	42.60 (±5.62)	43.14 (±7.15)
Self-selected gait speed (m/s)	0.79 (±0.36)	0.78 (±0.39)	0.79 (±0.36)

*Values presented are means with standard deviations in parentheses for continuous variables, or counts for categorical variables. LE, lower extremity; MMT, manual muscle testing; R, right; L, left*.

**Figure 2:**
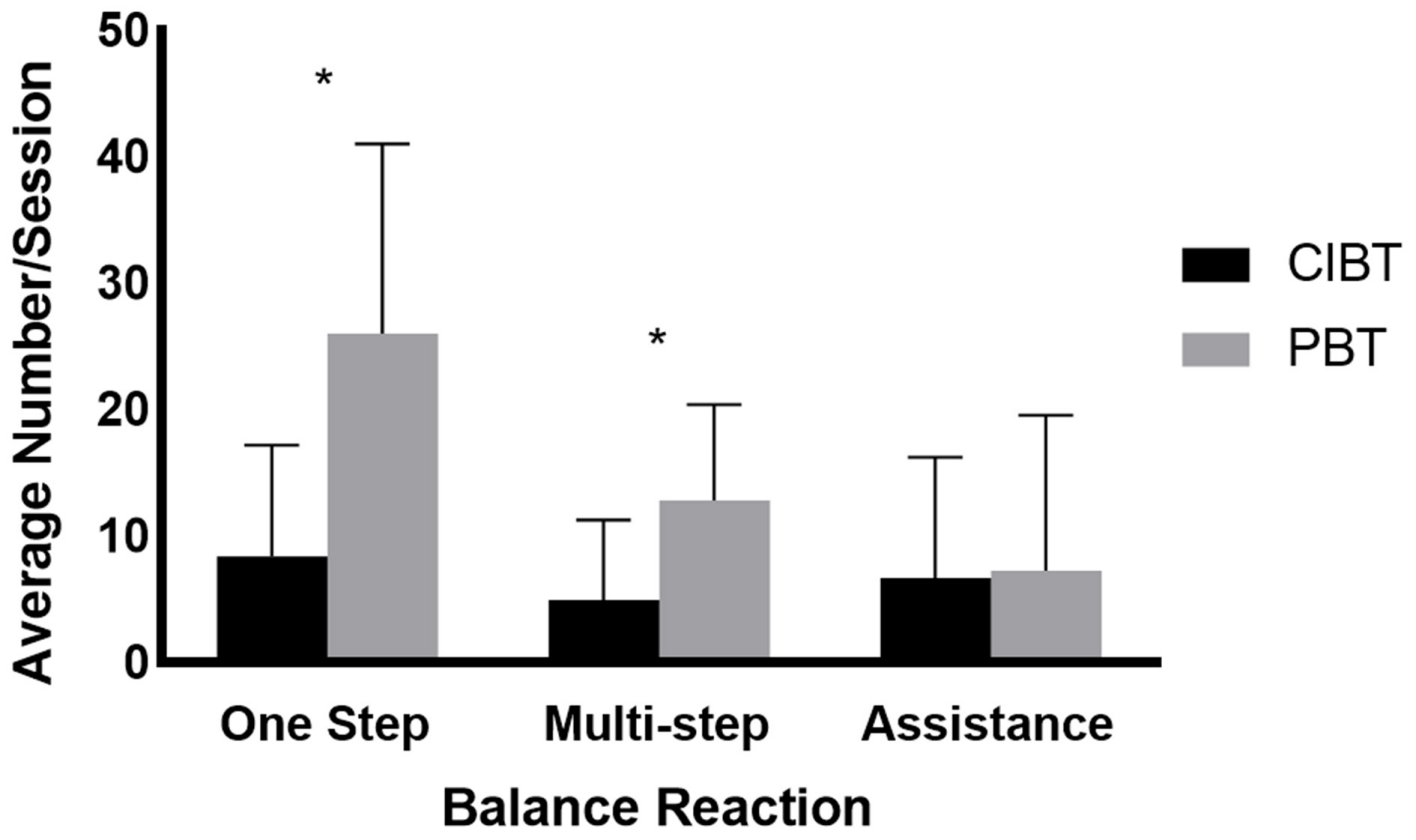
Balance reactions during training. The mean (standard deviation) number of balance reactions that occurred during training for both the PBT and CIBT groups. According to independent *t*-tests, the number of single step and multi-step reactions were different between the two groups, but there were no differences in the number of trials where assistance was needed to recover. *p < 0.05.

Participants in the PBT group experienced more single step responses (*t*_15_ = −3.21, *p* = 0.01) and multi-step (*t*_17_ = −2.51, *p* = 0.03), but not fall responses (*t*_17_ = −0.12, *p* = 0.41) during training (see Figure 2). There were no differences in challenge ratings over time (*F*_18_ = 0.39, *p* = 0.54), between groups (*F*_18_ = 1.43, *p* = 0.25), or any interaction effects (*F*_18_ = 0.07, *p* = 0.80). One participant in the PBT group began a new pulmonary rehabilitation program during participation in the study. There was one adverse event; a controlled fall during a training perturbation for a PBT participant who was practicing activities outside of the harness. No injuries resulted from this fall.

### Primary Outcome: Lean-and-Release Test

**Figure 3:**
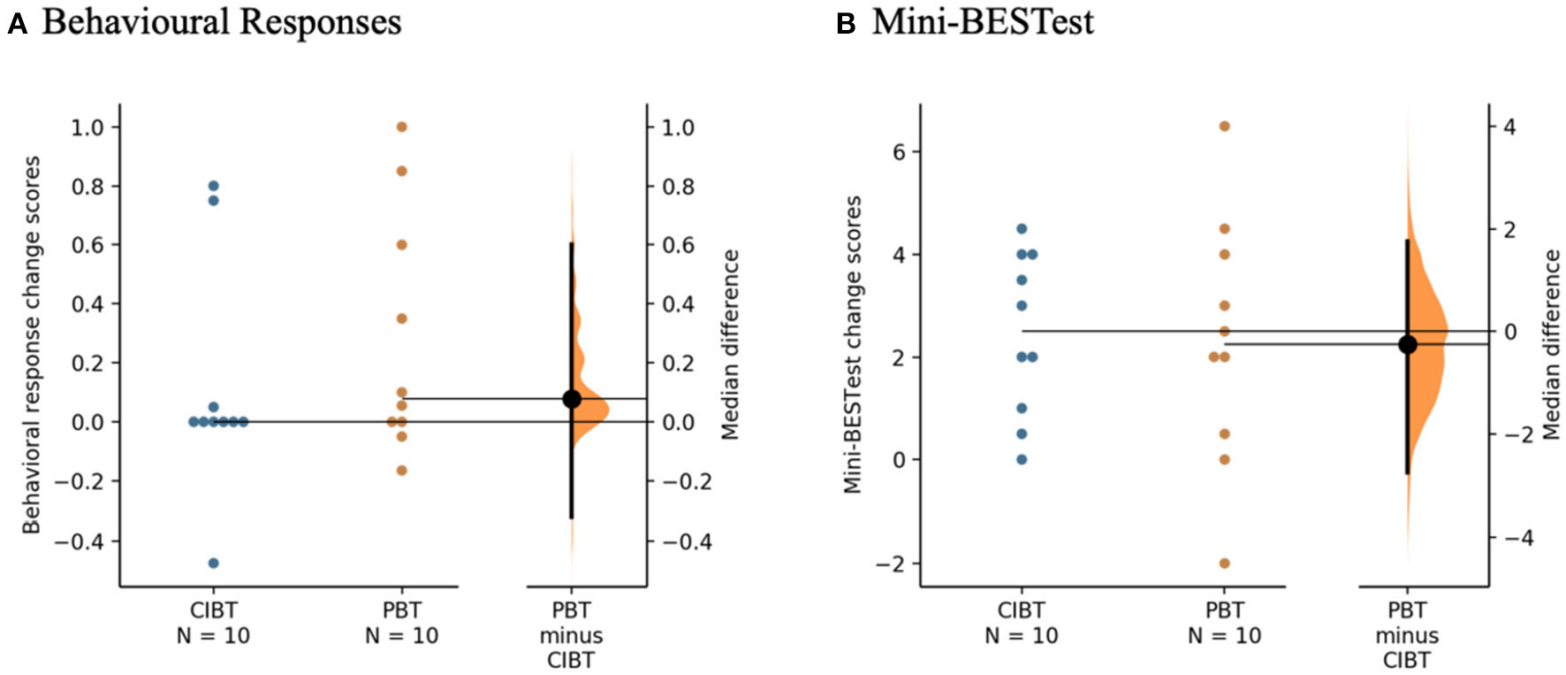
Gardner-Altman estimation plots for the clinical measures. **(A)** Behavioral Responses of the Lean-and-Release test and **(B)** Mini-BESTest. The median differences between the PBT and CIBT groups are represented in the Gardner-Altman estimation plots. Both groups' scores are plotted on the left axis and the median difference is plotted on the right axis as a bootstrap sampling distribution. The median difference and 95% confidence intervals are represented by the bold black dot and line, respectively. Figure developed from (50).

Median (interquartile range: IQR) change score in the behavioral response was 0.08 (0.68) for the PBT group and 0.00 (0.22) for the CIBT group (see Figure 3A). A mixed ANOVA showed no group or interaction effects (*F*_16_ = 0.58, *p* = 0.46, and *F*_16_ = 0.74, *p* = 0.40, respectively), but did show an effect of time (*F*_16_ = 4.55, *p* = 0.049). *Post hoc* tests did not show significant differences between the baseline average, 4-week or 8-week scores in any pairwise comparisons (*p* = 0.15->0.99). Chi Square tests of independence showed no differences between groups for the number of participants that achieved the MDC in the behavioral response from baseline to the final assessment (χ^2^ = 0.27, *p* = 0.61), with 2/10 CIBT participants and 3/10 PBT participants achieving an increase of at least 0.5 on the proportion of trials recovered with a single step.

Force plate data were available for 17 participants at the baseline and 8-week assessments and 15 participants at the 4-week assessment. Aside from the previously mentioned incomplete 4-week assessments, data were not available from participants unable to take any reactive steps during the Lean-and-Release test (*n* = 3, 2 PBT; 1 CIBT). Mean (standard deviation) change scores of foot contact time were −0.05 (0.08) s for the PBT group and 0.04 (0.12) s for the CIBT group. A mixed ANOVA showed no effects for time (*F*_13_ = 0.64, *p* = 0.44), group (*F*_13_ = 0.00, *p* = 0.97), or interaction (*F*_13_ = 2.41, *p* = 0.14).

### Secondary Outcomes: Balance, Strength, Gait, and Self-Report Measures

On the Mini-BESTest there was a median (IQR) change of 2.3 (3.8) points for the PBT group and 2.5 (3.1) points for the CIBT group (see Figure 3B). There was a significant effect of time (*F*_18_ = 26.78, *p* < 0.01), but no group or interaction effects (*F*_18_ = 0.67, *p* = 0.42 and *F*_18_ = 0.03, *p* = 0.87, respectively). *Post hoc* tests showed improvements from baseline to 4-week (*p* < 0.01) and baseline to 8-week (*p* < 0.01) but not between the 4-week and 8-week scores (*p* > 0.99). One participant in the PBT group improved beyond the MDC of five points. There were no differences between groups with respect to the proportion of participants who exceeded the MDC using Chi Square tests of independence (χ^2^ = 1.05, *p* = 0.31).

Only 13 participants (PBT *n* = 7, CIBT *n* = 6) were able to complete the CB&M, as this measure does not allow the use of gait aids. There was a mean (standard deviation) change of 5.8 (8.8) points for the PBT group and 4.5 (4.4) for the CIBT group. There were no group or interaction effects (*F*_18_ = 0.46, *p* = 0.51 and *F*_18_ = 0.17, *p* = 0.68, respectively), but there was a significant effect for time (*F*_18_ = 10.88, *p* = 0.04). *Post hoc* testing showed a difference between baseline and 4-week scores (*p* = 0.01) and baseline and 8-week scores (*p* = 0.02), but not between 4-week and 8-week scores (*p* > 0.99). Three PBT participants and one CIBT participant improved beyond the MDC, and there were no differences between groups with respect to the proportion of participants who exceeded the MDC (χ^2^ = 2.40, *p* = 0.12).

Median (IQR) change scores for lower extremity strength were 1.88 (5.81) points for the PBT group and 1.50 (6.19) points for the CIBT group. There were no significant findings for time (*F*_18_ = 4.33, *p* = 0.05), group (*F*_18_ = 0.00, *p* = 0.98), or interaction (*F*_18_ = 1.36, *p* = 0.26) effects.

The mean (standard deviation) changes in gait parameters are presented in Table 2. None of the gait parameters showed any effects for time, group, or interaction (see Table 2). No participants in either group improved beyond the MDC for walking speed or step length, and there were no between group differences for cadence (χ^2^ < 0.01, *p* > 0.99).

**Table 2:** Mean changes of gait parameters presented by group, as well as ANOVA values for time, group, and interaction effects.

**Gait parameter**	**PBT mean change (standard deviation)**	**CIBT mean change (standard deviation)**	**Time *F*_**15**_ (*p-*value)**	**Group*F*_**15**_ (*p-*value)**	**Interaction *F*_**15**_ (*p-*value)**
Step length (cm)	0.19 (3.39)	2.37 (3.55)	1.82 (0.20)	0.74 (0.40)	2.86 (0.11)
Walking speed (m/s)	0.00 (0.12)	0.05 (0.08)	1.02 (0.33)	0.03 (0.87)	1.21 (0.29)
Cadence (steps/min)	−0.60 (9.56)	0.51 (4.26)	0.02 (0.89)	0.27 (0.61)	0.06 (0.81)
Double support %	0.06 (2.61)	−1.18 (2.92)	1.23 (0.29)	0.01 (0.93)	1.07 (0.32)

On the ABC Scale, there was a mean (standard deviation) change score of 11.07 (11.36) for the PBT group and 11.52 (10.66) for the CIBT group. There were no significant group-by-time or group effects [*F*_(1,18)_ = 0.01, *p* = 0.93 and *F*_(1,18)_ = 0.06, *p* = 0.82, respectively], but there was a significant effect of time [*F*_(1,18)_ = 21.03, *p* < 0.00]. Three out of 10 PBT participants and four out of 10 CIBT participants improved their score beyond the MDC of 15% on the ABC Scale; there were no differences between groups with respect to the proportion of participants who exceeded the MDC (χ^2^ = 0.22, *p* = 0.64).

**Figure 4:**
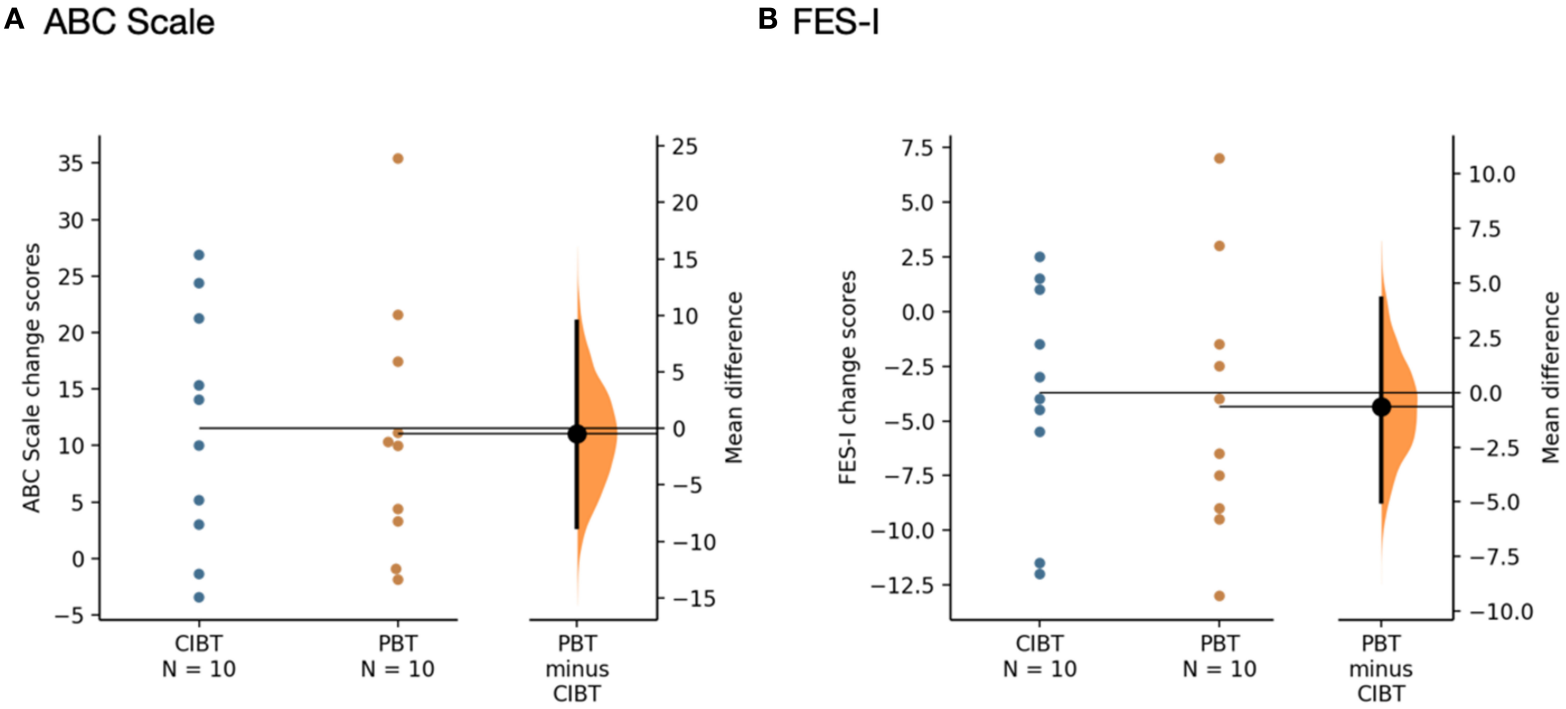
Gardner-Altman estimation plots for self-report measures **(A)** ABC Scale and **(B)** FES-I. The mean differences between the PBT and CIBT groups are shown in the Gardner-Altman estimation plots. Both groups' scores are plotted on the left axis and the mean difference is plotted on the right axis as a bootstrap sampling distribution. The mean difference and 95% confidence intervals are represented by the bold black dot and line, respectively. Figure developed from (50).

On the FES-I, mean (standard deviation) changes scores were −4.4 (6.1) for the PBT group and −3.7 (5.0) for the CIBT group. There were no significant group-by-time or group effects (*F*_(1,18)_ = 0.07, *p* = 0.80, *F*_(1,18)_ = 2.30, *p* = 0.15, respectively), but there was a significant effect for time (*F*_(1,18)_ = 10.44, *p* = 0.010). One participant out of 10 from the PBT and two out of 10 from the CIBT group moved below the cut-off score of 22 points; there was no difference between groups with respect to the proportion of participants who moved below the cut-off point (χ^2^ = 0.39, *p* = 0.53). Gardner-Altman estimation plots representing the change in self-report scores are shown in Figure 4.

### Follow Up

Of the 20 participants, 12 completed the 6-month assessment. Reasons for missing the assessment were significant changes in health (i.e., fracture *n* = 1, pregnancy *n* = 1, exacerbation of comorbid condition *n* = 2), and participants being unable to attend due to work schedules (*n* = 2) and caregiving/family commitments (*n* = 2). All participants except one (due to pregnancy) completed the 3-month assessment. Due to attrition at 6-months, we focused on the 3-month follow up scores to evaluate retention for the Lean-and-Release and clinical measures. All participants completed the self-report measures at follow up, except one participant who was unable to complete the measures at the 6 month follow up due to caregiving/family commitments. No falls data were lost during the 6 month follow up period, with all participants completing the interviews with the researcher as well as fall surveys after any falls.

At the 3-month assessment, all participants retained their gains as demonstrated by significant effects of time on the Lean-and-Release test behavioral response (*F*_15_ = 5.71, *p* = 0.03), Mini-BESTest (*F*_17_ = 9.00, *p* = 0.01), and FES-I (*F*_18_ = 9.44, *p* = 0.01). Retention on the ABC Scale did not reach statistical significance according to the ANOVA (*F*_18_ = 4.13, *p* = 0.06).

**Figure 5:**
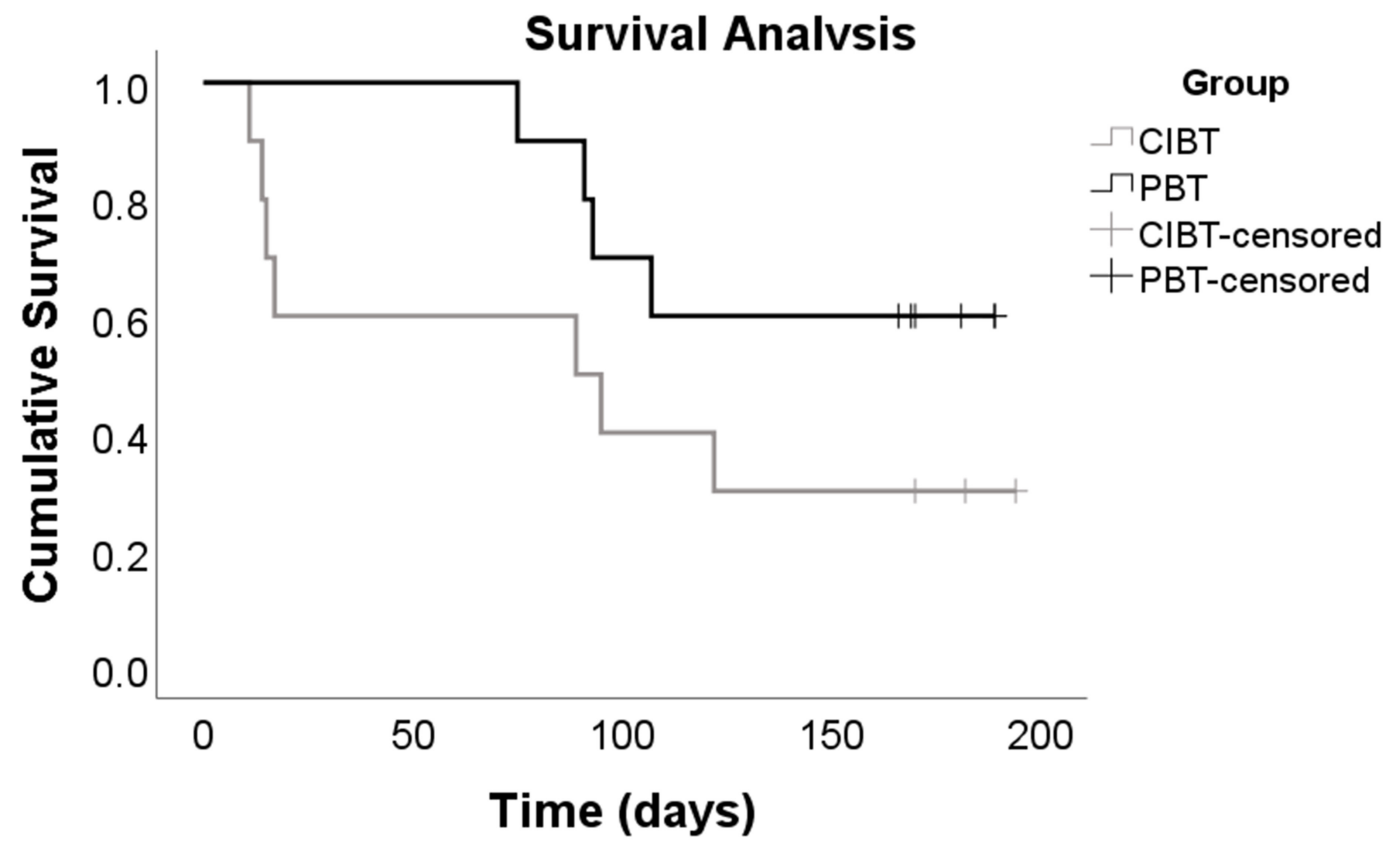
Kaplan-Meier Curve. This Kaplan-Meier curve represents the time to first fall for both the CIBT and PBT groups, participants who did not experience any falls during the 6 month follow up period are labeled as censored events. Although the *p*-value of 0.13 indicates there is no difference between groups, this curve shows the CIBT group experienced, on average, their first fall sooner than the PBT group.

During the 6 month follow up period, seven CIBT participants and four PBT participants experienced at least one fall; there was no between group difference for number of participants reporting at least one fall (χ^2^ = 1.82, *p* = 0.18). The relative risk for the PBT group was 0.54 (C.I. 0.22–1.35). There were a total of 31 falls (CIBT *n* = 21, PBT *n* = 10); despite the PBT group experiencing half as many falls as the CIBT group, the total number of falls was not significantly different between the two groups (*p* = 0.06). The incident rate of falls was 0.48 (C.I. 0.22–1.00) for the PBT group. Of all the participants, seven had more than one fall during the follow-up period (CIBT *n* = 4, PBT *n* = 3); there was no between group difference in the number of frequent fallers (χ^2^ = 0.27, *p* = 0.61). Seven of the 21 falls in the CIBT group, and two of the 10 falls in the PBT group resulted in injuries, which was not different between groups (χ^2^ = 1.5, *p* = 0.21); only one injury required medical attention due to a foot fracture from a fall outside at home (PBT participant). Most other injuries were bruises, cuts/scrapes, and pain. Mean time to first fall was 94.5 days (C.I. 48.0–140.0) for the CIBT group and 150.0 days (C.I 120.1–180.0) for the PBT group. There were no significant differences between groups for time to first fall according to the Kaplan-Meier analysis (χ^2^ = 2.31, *p* = 0.13), however the curve suggests a difference between the groups (see Figure 5). For those who fell, median (IQR) time to first fall was 17 (81) days for the CIBT group and 92 (25) days for the PBT group. The circumstances surrounding the falls, gathered by the falls survey, are presented in Table 3. Most falls occurred inside the home, during the daylight hours, and during standing or walking activities. The most common contributing factors reported for the falls were weakness, poor balance, or feeling tired.

**Table 3:** Data from the fall surveys are presented as numbers and percentages of responses for both the CIBT and PBT groups, as well as the total sample.

**Variable**		**CIBT *n* (%)**	**PBT *n* (%)**	**Total Sample *n* (%)**
Time of Day	Morning	9 (43%)	3 (30%)	12 (39%)
	Afternoon	7 (33%)	3 (30%)	10 (32%)
	Evening	4 (19%)	4 (40%)	8 (26%)
	Night	1 (5%)	0 (0%)	1 (3%)
Location	Home indoors	12 (57%)	6 (60%)	18 (58%)
	Community indoors	4 (19%)	2 (20%)	6 (19%)
	Community outdoors	5 (24%)	0 (0%)	5 (16%)
	Home outdoors	0 (0%)	2 (20%)	2 (6%)
Activity	Walking	9 (43%)	3 (30%)	12 (39%)
	Standing	7 (33%)	5 (50%)	12 (39%)
	Tub/shower transfer	1 (5%)	1 (10%)	2 (6%)
	Stairs	1 (5%)	0 (0%)	1 (3%)
	Changing positions	1 (5%)	0 (0%)	1 (3%)
	Opening/closing door	1 (5%)	0 (0%)	1 (3%)
	Sitting	0 (0%)	1 (10%)	1 (3%)
	Laying	1 (5%)	0 (0%)	1 (3%)
Contributing factors(Participants were able to select more than one option)	Weakness	8 (38%)	2 (20%)	10 (32%)
	Poor balance	6 (29%)	3 (30%)	9 (29%)
	Tired	7 (33%)	2 (20%)	9 (29%)
	Legs gave out	5 (24%)	3 (30%)	8 (26%)
	Dual task	3 (14%)	3 (30%)	6 (19%)
	Tripped	4 (19%)	2 (20%)	6 (19%)
	Slipped	4 (19%)	1 (10%)	5 (16%)
	Distracted	3 (14%)	1 (10%)	4 (13%)
	Not using assistive device	1 (5%)	2 (20%)	3 (10%)
	Rushing	3 (14%)	0 (0%)	3 (10%)
	Dark environment	1 (5%)	0 (0%)	1 (3%)

*Participants had to select one option from a multiple choice menu for time of day, location, and activity being performed at the time of the fall, however participants were able to select as many options as they wished to identify the contributing factors for the fall*.

## Discussion

Following PBT and CIBT, reactive stepping ability, balance control, balance confidence, and fall concern improved across the entire sample, suggesting that repetitive exposure to challenging balance training can lead to improvements regardless of the inclusion of external perturbations. Effects of the training programs were retained 3 months following training completion. When compared to CIBT, PBT may result in fewer falls and a longer time to first fall during a 6 month follow up period, although number of fallers does not appear to differ between groups. These results give us confidence that PBT is appropriate for individuals with chronic iSCI/D, although the use of a safety harness is imperative to avoid adverse events. Following the fall in a PBT participant, we only applied perturbations while participants were in the safety harness to avoid further adverse events.

These results contrast with what has been reported in other patient populations with neurological impairment, where PBT resulted in greater improvements in reactive stepping ability (51–53), clinical measures of balance (26, 27), and balance confidence (26) when compared to a frequency-matched control group. However, the participants in these studies were older adults (51–53), individuals with Parkinson's disease (26), and individuals who had experienced a stroke (27); none of whom had damage to their spinal cord. To our knowledge, the FES-I has only been evaluated following PBT in one study of older adults, where it was found to be unaffected by the training (54). This finding is also in conflict with our results, which showed a decrease in fall concern following both balance training programs. We suspect the reason our participants did not show any added benefit from the external perturbations may be because the spinal cord plays an important role in controlling postural reactions (31, 55). As our participants all had spinal cord impairment, it is possible that the neural mechanisms responsible for basic postural reflexes were inhibited, and therefore the capacity for improvement may be reduced due to the location of damage to the nervous system, since balance reactions are thought to originate in the spinal cord (30, 31). Individuals with damage in supraspinal areas, such as seen following stroke or Parkinson's disease, would have intact long-loop reflexes, whereas individuals with iSCI/D may not. Further research is warranted to investigate if the level and/or location of damage to the spinal cord affect reactive balance control and capacity for its improvement. Another possible reason for these discrepancies could be because individuals with iSCI/D typically receive little time for balance training during rehabilitation (5), and therefore any increase in balance training may lead to participants reporting improvements in these constructs. We are not surprised, however, that foot contact times did not change following training, as recent research has shown that individuals with iSCI/D do not demonstrate differences in this parameter when compared to individuals without SCI/D (21).

Our findings are also in contrast with some previous research that demonstrated improvements in gait parameters and lower extremity strength after balance training in people with iSCI/D, however previous research was done using anticipatory balance training. Using visual feedback to augment standing balance training, Tamburella et al. found improvements in gait parameters, including speed, cadence, stride length, and time spent in double support (15). They also demonstrated that improvements in static balance measures, such as postural sway, appeared before improvements in gait parameters, indicating the importance of balance for ambulation (15). A study using virtual reality to train balance found improvements in speed and stride length, but not stride frequency (56). Virtual reality-based balance training has also been shown to improve lower extremity strength in individuals with iSCI/D when five muscle groups were tested manually (16, 17). A case study examining the effects of a divided attention stepping task, however, did not find any effects on lower extremity strength following training, but this testing was done with a dynamometer on three muscle groups (18). Our results may differ from these findings for several reasons. The participants in these studies were stronger at baseline than many of our participants, indicating a need for future research to include participants with varying levels of impairment. While one study targeted a stepping task (18), the studies using virtual reality targeted different lower extremity movements, such as ankle dorsiflexion and hip abduction/adduction (16, 17).

Participants were as likely to fall during standing as during walking, which is different than in previously reported studies, where more falls in people with iSCI/D occur while walking (57–59). However, the participants in these studies were all full-time ambulators, while some participants in our study used a wheelchair as their primary means of mobility, and previous research has shown that walking is not commonly associated with falls for this group (60). It is likely that our findings reflect the common activities associated with falls for both ambulators and wheelchair users. The between group difference in the number of fallers was not statistically significant, but this could be attributable to a small sample size. Our findings must viewed as exploratory, and powered studies must take place to confirm our results. We found that, compared with CIBT participants, PBT participants had a relative risk of 0.54 to experience a fall during the follow up period, although this difference was not statistically significant. A recent systematic review found that PBT resulted in fewer fallers than control interventions for individuals with Parkinson's disease and older adults, with a risk ratio of 0.71 (23). Another study in individuals who had experienced a stroke found that, when compared with a historical control group, fewer PBT participants fell, with a risk ratio of 0.21 (29).

The CIBT group experienced twice as many falls as the PBT group during the 6-month follow up period, results which are similar to previous research. Of note, two of the three participants who experienced five or more falls during the follow up period were in the CIBT group. The previously mentioned systematic review found a rate ratio of 0.54 when viewing fall rates following PBT training compared to control groups (23), which is very similar to our incident rate of 0.48. Time to first fall is a variable that has rarely been calculated in other PBT studies, but our exploratory findings indicate that this is a noteworthy outcome. The Kaplan-Meier curve (Figure 5) suggests a difference between the groups, and the lack of significance is likely due to the sample size. A possible reason for the observed difference between groups may be that the individuals in the PBT group were better prepared to recover their balance after repeated practice during the training program, and therefore did not fall even when balance was perturbed in their daily lives. The PBT group experienced losses of balance due to both internally and externally generated perturbations, which both contribute to fall risk. The CIBT program focused mainly on anticipatory balance challenges, and although participants in this group did experience losses of balance during training as well, they were only required to recover from internally generated perturbations. The addition of externally generated perturbations may mimic real-world challenges, such as slips and trips, which may explain why PBT participants did not fall as soon as CIBT participants post-training. It is also possible that the longer time to first fall indicates that effects of the PBT program wore off throughout the 6-month follow up period; if this was the case, “booster sessions” could be used occasionally to allow individuals to practice reactive stepping ability.

It is also possible that our sample's heterogeneity in balance ability affected the results. Our inclusion criteria were broad, as this study is the first of its kind in the iSCI/D population. Participants ranged in mobility status, level of injury, and baseline scores, which could have impacted our findings. Of note, more participants in the CIBT group had an injury to the cervical spine than in the PBT group, which could influence motor synergies and reactive control. Some participants showed significant improvements in reactive stepping ability as well as clinical balance scores regardless of group allocation; typically, the participants who were able to ambulate without a gait aid. When looking at lower extremity muscle strength, however, there were no differences between individuals who demonstrated improvements in reactive stepping and those who did not. It is plausible that some level of dynamic balance control is necessary before implementing challenging programs such as CIBT or PBT. Furthermore, it is possible that PBT is most effective for individuals who are able to initiate a step without upper extremity support. To participate in PBT, individuals who are unable to step without a gait aid may benefit from methods to facilitate stepping, such as functional electrical stimulation (FES).

### Limitations

One limitation is our small sample for the secondary outcomes; results are only powered for the primary outcome. However, our *ad hoc* interim analysis demonstrated that treatment effects would only be seen with an impractical sample size when looking at the primary outcome. Furthermore, we recruited using a convenience sampling method, and used broad inclusion criteria, which could have affected our results. The statistical measures used to examine the effects of time were *post hoc* analyses and not determined *a priori* (32). Secondly, we experienced a significant loss to follow up at the 6 month time point. Third, any medications being taken by our participants could have influenced our results; however, we did not track medication usage. Fourth, the CB&M was not appropriate for our entire sample, as not all participants were able to complete the assessment. Including a measure of standing balance may have been more appropriate for some participants. Fifth, it is possible that not all fall surveys were completed within 24 h of the occurrence of the fall, increasing the likelihood that responses were affected by recall bias, and falls were not tracked prior to participation in the study, limiting our knowledge of participants' falls history. Finally, the FES-I has not been validated for use in individuals with iSCI/D, although it has been used in studies with this population before (61).

## Conclusions

PBT does not appear to be more effective than CIBT to improve reactive stepping ability, balance, strength, gait, or self-reported balance confidence or fall concern for individuals with chronic iSCI/D; however, participants showed improvement over time by participating in intensive and challenging balance training programs, regardless of exposure to external balance perturbations. These findings indicate that increased time (i.e., 24 h) spent on balance training during the chronic phase of iSCI/D may improve balance ability. Furthermore, these findings indicate that PBT may be more effective than CIBT to decrease fall rates and increase the time to first fall for individuals with iSCI/D. Future work is warranted to investigate the impact of PBT on fall-related outcomes and to evaluate the effects of balance training in a subacute and/or inpatient population.

## Data Availability Statement

The raw data supporting the conclusions of this article will be made available by the authors, without undue reservation.

## Ethics Statement

The studies involving human participants were reviewed and approved by Research Ethics Board of the University Health Network. The patients/participants provided their written informed consent to participate in this study.

## Author Contributions

JU, BC, AM, KM, and KEM designed the study. JU, KC, JL, and KEM collected the data. JU, KC, JL, MA, and KEM analyzed the data. JU, KC, JL, BC, AM, MA, KM, and KEM contributed to the manuscript. All authors contributed to the article and approved the submitted version.

## Conflict of Interest

The authors declare that the research was conducted in the absence of any commercial or financial relationships that could be construed as a potential conflict of interest.
